# A bivalent mRNA–LNP vaccine confers broad-spectrum protection against both homologous and heterologous H5/H7 highly pathogenic avian influenza viruses in SPF chickens

**DOI:** 10.1186/s13567-026-01790-2

**Published:** 2026-06-18

**Authors:** Dandan Wei, Yu Pan, Xinkui Zhang, Yun Quan, Simin Feng, Mengting Huang, Lei Sun, Yujia Yang, Jiaji Zhou, Xinyu Han, Beibei Niu, Qiong Zhang, Weixin Jia

**Affiliations:** 1 Guangdong Engineering Laboratory for Medicament of Zoonosis Prevention and Control, Key Laboratory of Zoonoses Prevention and Control of Guangdong Province, National Avian Influenza Para-Reference Laboratory (Guangzhou), College of Veterinary Medicine, South China Agricultural University, Guangzhou, China; 2 Guangzhou National Laboratory, Guangzhou, China; 3 Engineering Research Center for the Prevention and Control of Animal Original Zoonosis of Fujian Province University, College of Life Science, Longyan University, Longyan, 364012 China; 4 State Key Laboratory of Respiratory Disease, National Clinical Research Center for Respiratory Disease, Guangzhou Institute of Respiratory Health, The First Affiliated Hospital of Guangzhou Medical University, Guangzhou, China; 5 Key Laboratory of Virology and Biosafety, Wuhan Institute of Virology, Chinese Academy of Sciences, Wuhan, Hubei China

**Keywords:** Avian influenza, mRNA vaccine, H5N1, H7N9, broad-spectrum protection, transcriptome

## Abstract

The continuous evolution and co-circulation of H5 and H7 subtype highly pathogenic avian influenza viruses (HPAIVs) have caused substantial economic losses to the global poultry industry and pose a persistent threat to public health. This study aimed to develop a bivalent nucleoside-modified messenger (mRNA) vaccine encoding the hemagglutinin (HA) antigens of circulating H5N1 and H7N9 strains and to evaluate its immunogenicity and protective efficacy in an SPF chicken model. The bivalent vaccine elicited robust humoral immunity in SPF chickens in a dose-dependent manner following immunization. The immune sera exhibited potent neutralizing activity against the homologous viruses and cross-reactivity with heterologous strains. Challenge experiments demonstrated that chickens immunized with high doses (50 μg and 80 μg) of the bivalent mRNA vaccine were 100% protected against lethal challenge with homologous and heterologous H5N1, as well as heterologous H7N9 viruses. Furthermore, the vaccine effectively suppressed viral replication in the lungs and significantly reduced or blocked viral shedding. Importantly, splenic transcriptome sequencing revealed that vaccination elicited extensive immune reprogramming, with marked upregulation of key genes associated with Th1-type immune responses, antigen presentation, and cytokine production. The bivalent mRNA–lipid nanoparticle (LNP) vaccine developed in this study exhibited excellent immunogenicity and broad-spectrum protective potential in SPF chickens. Transcriptomic analysis further elucidated the molecular mechanisms underlying the vaccine-induced protective immunity. This study provides a promising vaccine candidate for the control of the co-circulation of H5 and H7 subtype HPAIVs.

**Supplementary Information:**

The online version contains Supplementary Material available at 10.1186/s13567-026-01790-2.

## Introduction

H5 and H7 subtype highly pathogenic avian influenza viruses (HPAIVs) are widely recognized as major threats to the poultry industry and critical hazards to public health security, attributable to their high mortality rates, rapid transmission dynamics, and inherent potential for cross-species spillover. In recent years, the clade 2.3.4.4b H5N1 HPAIV has undergone explosive global expansion, exacerbating the already pressing epidemiological burden. Since October 2023, a cumulative total of 2749 outbreaks has been officially reported to the World Organisation for Animal Health (WOAH, as of the time of writing), leading to the death or culling of hundreds of millions of poultry worldwide [1]. Furthermore, frequent spillover infections into wild birds and mammalian species have also been reported [2–4], with 112 human cases of H5N1 infection reported to the World Health Organization (WHO) [5, 6]. Despite the implementation of vaccination programs that have partially controlled H7N9 in poultry populations, circulating H7N9 variants continue to evolve [7, 8]. Historically, H7N9 has caused five severe waves of human infection; since early 2013, 1568 laboratory-confirmed human cases of H7N9 have been reported to the WHO, including 616 deaths [9], underscoring a non-negligible public health risk.

The co-circulation of H5 and H7 viruses in the field not only increases the risk of coinfection in poultry but also facilitates genetic re-assortment, potentially leading to the emergence of novel variants with enhanced pathogenicity and transmissibility. Within the “One Health” framework, vaccination of poultry against H5 and H7 subtype HPAIVs remains a central component of integrated control strategies. Since 2017, the implementation of H5/H7 bivalent or trivalent vaccination programs in China has substantially reduced the circulation of H7N9 in poultry and eliminated reported human infections [10–12]. Nevertheless, sporadic outbreaks caused by multiple HPAIV subtypes, including H5N1 [13], H5N6 [14], H5N8 [15], and H7N9 [16], continue to be detected in poultry flocks.

Although inactivated vaccines remain a cornerstone of HPAI prevention, their utility is constrained by inherent limitations, including laborious production processes, suboptimal induction of cellular immunity, and biosafety concerns associated with manufacturing. As an advanced vaccine platform, messenger RNA (mRNA) vaccines have demonstrated exceptional efficacy in combating the coronavirus disease 2019 (COVID-19) pandemic [17, 18]. Compared with conventional vaccine platforms, mRNA vaccines rely on cell-free synthesis [19], facilitating rapid large-scale production and flexible sequence updates to counter emerging variants, such as severe acute respiratory syndrome coronavirus 2 (SARS-CoV-2) [17, 20, 21] and influenza viruses [22, 23]. While these mRNA vaccines have been clinically validated in humans, the application of mRNA vaccines in the veterinary sector remains nascent, particularly for the prevention and control of avian influenza [24, 25].

In this study, we developed a bivalent nucleoside-modified mRNA–lipid nanoparticle (LNP) vaccine targeting the currently circulating HPAI H5N1 (clade 2.3.4.4b) [14] and H7N9 (group y.2.4) [7] strains. Specifically, the vaccine comprises LNP-encapsulated mRNAs that individually encode the HA proteins of the H5N1 and H7N9 subtypes. We evaluated the immunogenicity and protective efficacy of this bivalent mRNA–LNP vaccine in specific-pathogen-free (SPF) chickens following lethal challenge with homologous and heterologous H5 and H7 HPAIVs. Additionally, by integrating transcriptomic sequencing, we analyzed the gene expression profile changes in the spleens of immunized SPF chickens, thereby elucidating the molecular mechanisms underlying the protective immunity induced by the mRNA vaccine. Collectively, this study provides experimental evidence supporting the development of next-generation mRNA-based vaccines for the prevention and control of avian influenza.

## Materials and methods

### Biosafety and ethics statement

All experiments involving live H5 and H7 subtype HPAIVs and challenge studies in SPF chickens were performed in the biosafety level 3 (BSL-3) and animal biosafety level 3 (ABSL-3) facilities at South China Agricultural University (SCAU, Guangzhou, China; CNAS biosafety certification no. BL0011). All animal care and experimental procedures were conducted in strict accordance with the protocols approved by the Institutional Animal Care and Use Committee (IACUC) of SCAU.

### Cells and viruses

BHK-21, DF-1, and MDCK cells were maintained in Dulbecco’s modified Eagle medium (DMEM) supplemented with 10% fetal bovine serum (FBS) and 1% penicillin–streptomycin at 37 ℃ with 5% CO_2_. SPF chicken embryos, aged 9–11 days, were purchased from Guangdong Wen’s Dahuanong Biotechnology Co., Ltd. (Yunfu, China). The HPAIV strains used in this study included A/Duck/Guangxi/B1417/231129/H5N1 (hereafter referred to as H5N1/B1417), A/Chicken/Guangdong/B468/240419/H5N1 (H5N1/B468), A/Duck/Shandong/B1338-10/231116/H5N6 (H5N6/B1338-10), A/Chicken/HeB/363–4/2022/H7N9 (H7N9/363–4), and A/Chicken/HeB/257–3/2022/H7N9 (H7N9/257–3). Viruses were propagated in SPF chicken embryos at 37 ℃ for 36–72 h. Subsequently, the infected embryos were chilled at 4 ℃ overnight. The allantoic fluid was then harvested and centrifuged at 3000 *g* for 10 min at 4 ℃. The resulting supernatant was aliquoted and stored at −80 ℃.

### mRNA synthesis

The HA genes of HPAIV strains H5N1/B1417 and H7N9/363–4 were codon-optimized and individually cloned into mRNA production plasmid vectors containing a T7 promoter and the 5′ and 3′ untranslated regions (UTRs) of human *β-globin*. Additionally, Flag tag sequence was appended to the 3′ end of the HA coding sequence to facilitate subsequent protein detection. Linearized DNA templates containing a poly(A) tail were generated by polymerase chain reaction (PCR) amplification and purified using a PCR purification kit. These purified templates were then subjected to in vitro transcription (IVT) to synthesize H5-HA and H7-HA mRNAs incorporating N1-methylpseudouridine (m1Ψ) and 5′ cap structure. Following DNase I digestion to remove the template DNA, the mRNAs were purified via lithium chloride (LiCl) precipitation. Finally, mRNA integrity and concentration were assessed by TBE agarose gel electrophoresis, capillary electrophoresis (Agilent 5200), and NanoDrop spectrophotometry (NanoDrop One), respectively, prior to storage at −80 ℃.

### Cell transfection and expression verification

BHK-21 and DF-1 cells were transfected with the mRNA constructs to evaluate antigen expression, with the latter serving as a representative avian cell model. Transfections were performed using the Lipo8000 Transfection Reagent (Beyotime, Shanghai, China) following the manufacturer’s instructions. Briefly, cells were seeded into culture plates at a density of 2.5 × 10^5^ cells/mL. Upon reaching approximately 80% confluence, the cells were transfected with the respective mRNA constructs using Lipo8000.

Western blot analysis was performed to verify the expression of the mRNA-encoded proteins. Cells seeded in 12-well plates were transfected and cultured for 24 h. Total cellular proteins were harvested, separated by sodium dodecyl sulfate–polyacrylamide gel electrophoresis (SDS–PAGE) and transferred onto polyvinylidene fluoride (PVDF) membranes. The membranes were incubated with an anti-Flag polyclonal antibody and an anti-GAPDH monoclonal antibody, followed by incubation with the corresponding horseradish peroxidase (HRP)-conjugated secondary antibodies. Finally, protein bands were visualized using an enhanced chemiluminescence (ECL) substrate, and images were acquired with the iBright™ CL750 Imaging System. Additionally, both the cell culture supernatant and whole cell lysate were collected from the mRNA-transfected BHK-21 cells. The supernatant was concentrated 16-fold and subjected to western blot analysis, as described previously.

Antigen expression was further assessed by an indirect immunofluorescence assay (IFA). Cells seeded in 96-well plates were transfected as described above. At 24 h post-transfection, the cells were fixed with 4% paraformaldehyde (PFA) and either permeabilized with 0.2% Triton X-100 (for total protein expression) or left unpermeabilized (for membrane surface localization). Subsequently, the cells were incubated with the primary antibody MEDI8852 [26], a broad-spectrum antibody targeting the HA stem region, followed by an Alexa Fluor 488-conjugated goat anti-human IgG secondary antibody. Finally, images were acquired using an inverted fluorescence microscope to visualize HA expression. To evaluate the cellular localization of the protein, cells were left unpermeabilized and stained with DAPI to visualize nuclear morphology. The remaining steps were performed as described above. Finally, images were acquired using a ZEISS LSM880 NLO with Airyscan two-photon laser scanning confocal microscope.

Additionally, to quantitatively confirm the cell surface expression of the HA antigens, flow cytometry was performed. At 24 h post-transfection, cells were detached and harvested using phosphate-buffered saline (PBS) containing 5 mM EDTA, washed, and stained with the primary antibody MEDI8852 (without permeabilization) followed by an Alexa Fluor 488-conjugated secondary antibody. The fluorescence intensity of the cells was then analyzed using a flow cytometer. Finally, flow cytometry data were analyzed using FlowJo software.

### Preparation and characterization of LNP

The lipids were prepared by dissolving SM102, 1,2-distearoyl-*sn*-glycero-3-phosphocholine (DSPC), cholesterol, and PEG2000-DMG in 100% ethanol at a molar ratio of 50:10:38.5:1.5. H5-HA mRNA and H7-HA mRNA were mixed at an equal mass ratio in 50 mM sodium acetate buffer (pH 4.0). Subsequently, the mRNA mixture was encapsulated into LNPs using microfluidic technology in a volume ratio of 3:1 at a flow rate of 12 mL/min.

The mRNA–LNP solution was dialyzed against cold PBS to a final pH of 7. The particle size and polydispersity index (PDI) of the bivalent mRNA–LNP vaccine were measured at 25 ℃ by dynamic light scattering (DLS) using a Zetasizer Nano ZS (Malvern Panalytical). The zeta potential was also measured using the same instrument to evaluate the surface charge of the LNPs. Concurrently, the encapsulation efficiency (EE) and mRNA concentration were quantified using the Quant-iT RiboGreen assay (Thermo Fisher Scientific). Finally, the mRNA–LNP vaccines were concentrated to the desired concentration using Amicon Ultra-15 centrifugal filter units (Millipore Sigma). To ensure stability, the concentrated bivalent mRNA–LNP vaccine was formulated with a 6× sucrose cryoprotectant, aliquoted, and maintained at −80 ℃. The vials were thawed on ice immediately prior to immunization.

### Cryo-electron microscopy (Cryo-EM) characterization of mRNA–LNP

Morphological characterization of the bivalent mRNA–LNP vaccine was conducted utilizing cryo-EM. Initially, Quantifoil R1.2/1.3 holey carbon gold grids (300 mesh) overlaid with a 2-nm ultrathin carbon film were subjected to glow discharge. Subsequently, 3 μL of the mRNA–LNP suspension was deposited onto the treated grids and allowed to settle at ambient temperature for 1 min. Specimen vitrification was executed using a Vitrobot Mark IV apparatus (Thermo Fisher Scientific) under controlled environmental conditions (4 ℃ and 100% relative humidity); the grids were blotted for 3 s before being flash-frozen in liquid ethane. The resulting cryo-grids were imaged on a Titan Krios G4 transmission electron microscope (Thermo Fisher Scientific) operating at an accelerating voltage of 300 kV. The microscope was configured with a Falcon 4i direct electron detector coupled to a Selectris-X energy filter (10 eV slit). Automated data acquisition was managed by EPU2 software in counting mode, with imaging parameters set to a defocus range of −3 to −4 μm, a pixel size of 1.94 Å, and an accumulated electron dose of 20 e/Å^2^.

### SPF chicken experiments

SPF Babcock chickens, aged 3 weeks, were purchased from Jinan SPF Poultry Co., Ltd. (Jinan, China) and housed in SPF isolators (Fengshi Laboratory Animal Equipment Co., Ltd., Suzhou, China) during the immunization period. Environmental conditions within the isolators were strictly controlled, with the temperature maintained at 26 ± 2 ℃ using heat lamps, relative humidity at 60 ± 10%, and negative pressure at 100 ± 10 Pa. A light/dark cycle was established with light provided from 09:00 to 17:00 and darkness from 17:00 to 09:00. Feed and water were provided ad libitum.

SPF chickens were randomly assigned to four groups (*n* = 10 per group): three treatment groups receiving 20 μg (10 μg of each mRNA), 50 μg (25 μg of each mRNA), or 80 μg (40 μg of each mRNA) of the bivalent mRNA–LNP vaccine, and a PBS placebo control group. As outlined in Figure 2A, all chickens were immunized via intramuscular (IM) injection following a prime-boost regimen with a 2-week interval, while the control group received an equal volume of PBS. Blood samples were collected from the wing vein at 2 weeks after each immunization. After allowing the samples to clot at room temperature, serum was separated by centrifugation, aliquoted, and stored at −80 ℃ for subsequent serological analysis.

Then, 1 week after the booster immunization, three chickens from each group were euthanized. Spleens were harvested and placed in PBS, as outlined in Figure 3A. The tissues were mechanically dissociated under aseptic conditions, and single lymphocytes were isolated using a Chicken Spleen Lymphocyte Isolation Kit (Solarbio, Beijing, China) according to the manufacturer’s instructions. Briefly, the spleen cell suspension was carefully layered over an equal volume of separation medium, ensuring a distinct interface. Centrifugation was performed at 700 *g* for 25 min at room temperature using a swing-out rotor. Following centrifugation, the opaque lymphocyte layer at the interface was collected into a new tube and washed twice with washing buffer (250 *g*, 10 min). Finally, the supernatant was discarded, and the cells were either resuspended in Roswell Park Memorial Institute (RPMI)−1640 medium supplemented with 10% FBS and 1% penicillin–streptomycin for ELISpot assays or cryopreserved in freezing medium at −80 ℃ for transcriptomic sequencing.

Subsequently, 2 weeks post-booster immunization, viral challenge experiments were conducted. As illustrated in Figure 4A, SPF chickens in each group were intranasally challenged with 200 µL of 10^6^ 50% egg-infective dose (EID_50_) of one of the following viruses: the vaccine-matched (homologous) H5N1/B1417 strain, the heterologous clade 2.3.4.4 h H5N1/B468 strain, or the heterologous group y.2.3 H7N9/257–3 strain. To evaluate viral shedding, oropharyngeal and cloacal swabs were collected at 3 and 5 days post-challenge (dpc). Three chickens per group were euthanized on 3 dpc to harvest lung tissues, which were homogenized for viral load determination. Lungs were also immediately harvested from chickens that succumbed within 2 dpc for analysis. The remaining chickens were monitored for survival, and all survivors were euthanized at 14 dpc to conclude the experiment.

### Virus detection in SPF chicken embryos

Viral loads in lung tissues and viable virus in swab samples were determined using 9–11-day-old SPF chicken embryos. Lung tissues were homogenized in sterile PBS (0.1 g tissue per 0.1 mL) using a low-temperature tissue grinder. After low-speed centrifugation, the supernatant was serially diluted tenfold in PBS containing antibiotics and inoculated into the allantoic cavity of the SPF chicken embryos. Embryos were incubated at 37 ℃ for 36–72 h. For swab samples, supernatants were collected after centrifugation and inoculated as described above. Allantoic fluid samples exhibiting hemagglutination activity were considered virus-positive. The viral titers in lung tissues were calculated using the Reed–Muench method and expressed as the EID_50_. The viral shedding rate was defined as the percentage of virus-positive chickens among the total survivors at each time point.

### Enzyme-linked immunosorbent assay (ELISA)

Binding antibody titers against recombinant HA (rHA) in serum samples from immunized SPF chickens were determined by ELISA, as previously described [27]. Briefly, 96-well plates were coated overnight at 4 ℃ with 0.5 μg/mL of H5N1/B1417-rHA or H7N9/363–4-rHA in carbonate/bicarbonate coating buffer (pH 9.6). After washing three times with PBS, the plates were blocked with 5% skim milk in PBS at 37 ℃ for 1 h. The plates were incubated at 37 ℃ for 1 h with threefold serial dilutions of immune sera starting at 1:100. After washing with PBST, HRP-conjugated goat anti-chicken IgG secondary antibody was added and incubated at 37 ℃ for 1 h. Following final washes, colorimetric detection was performed utilizing 3,3′,5,5′-tetramethylbenzidine (TMB) substrate (Beyotime). The colorimetric reaction was terminated by the addition of an equal volume of stop solution and the absorbance recorded at 450 nm (OD_450_) using a PerkinElmer EnSight. Serum endpoint titers were defined as the highest serum dilution resulting in an OD_450_ value at least twofold higher than the background. All titers were log_10_-transformed prior to statistical analysis.

### Microneutralization (MN) assay

The neutralizing activity of immune sera against H5 or H7N9 viruses was evaluated using the fixed virus/diluted sera method. Prior to the assay, MDCK cells were seeded in 96-well plates at a density of 3 × 10^4^ cells per well and cultured for 24 h. Chicken sera were treated with receptor-destroying enzyme (RDE; Denka-Seiken) at a ratio of 1:3 (vol/vol), incubated at 37 ℃ for 16 h, and subsequently heat-inactivated at 56 ℃ for 30 min. The treated sera were serially diluted threefold (starting at 1:24) in DMEM supplemented with 25 mM HEPES, 1% penicillin–streptomycin, and 1 μg/mL TPCK-treated trypsin. The diluted sera were then mixed with an equal volume of viral suspension containing 100 focus forming units (FFU) of the respective viruses (homologous: H5N1/B1417, H7N9/363–4; heterologous: H5N6/1338–10, H5N1/B468, H7N9/257–3) and incubated at 37 ℃ for 1 h. The serum–virus mixtures were transferred to the MDCK cell monolayers and incubated at 37 ℃ for 1 h to allow adsorption. The mixtures were then aspirated, and the cells were washed with PBS. Subsequently, the cells were overlaid with minimum essential medium (MEM) containing 1% carboxymethyl cellulose (CMC), 1 μg/mL TPCK-treated trypsin, and 25 mM HEPES. Finally, the plates were incubated at 37 ℃ for 24–48 h. The number of viral spots in MDCK cells was determined by a virus formation assay. Briefly, cells were fixed with 4% paraformaldehyde for 1 h at room temperature, followed by permeabilization and blocking with PBS containing 1% bovine serum albumin (BSA) and 0.2% Triton X-100 for 30 min. The cells were then incubated sequentially with a mouse anti-NP monoclonal antibody (clone 4R5) and an HRP-conjugated goat anti-mouse IgG secondary antibody for 1 h each at 37 ℃. Viral foci were visualized using TrueBlue TMB substrate and counted with a CTL ImmunoSpot Analyzer. The 50% neutralizing antibody titer (MNT_50_) was defined as the reciprocal of the highest serum dilution that inhibited 50% of viral focus formation (relative to the virus control). Titers were log_10_-transformed for analysis.

### Hemagglutination inhibition (HI)

Chicken sera were serially diluted twofold in PBS in 96-well V-bottom plates. The diluted sera were mixed with an equal volume of inactivated virus containing 4 hemagglutination units (HAU) (identical to the strains used in the MN assay) and incubated at room temperature for 30 min. Subsequently, a 1% chicken red blood cell (RBC) suspension was added, followed by incubation for an additional 30 min. The HI titer was defined as the reciprocal of the highest serum dilution that completely inhibited hemagglutination. Titers were log_2_-transformed for analysis.

### Enzyme-linked immunospot (ELISpot)

Splenocytes were diluted to 2 × 10^6^ cells/mL. A 100-μL aliquot of the cell suspension was seeded into 96-well IFN-γ ELISpot plates (ELISpot Plus: Chicken IFN-γ HRP; Mabtech) and stimulated with 20 μg/mL of H5N1/B1417-rHA or H7N9/363–4-rHA protein for 48 h. RPMI-1640 medium alone served as the background control, while concanavalin A (ConA, 1 μg/mL) was included as a positive control. Subsequent steps were performed according to the manufacturer’s instructions. The cells were removed by washing, and the plates were incubated sequentially with the MT7C10-biotin detection antibody and streptavidin-HRP at room temperature. Spots were visualized using TMB substrate solution. After stopping the reaction, the plates were analyzed using a CTL ImmunoSpot Analyzer.

### RNA extraction, library construction, and transcriptome sequencing

Total RNA was extracted from frozen splenocytes using the Total RNA Extractor (Trizol) kit according to the manufacturer’s instructions. The RNA samples were subsequently treated with RNase-free DNase I to remove genomic DNA contamination. The integrity, purity, and concentration of the extracted RNA were assessed using agarose gel electrophoresis, a NanoPhotometer^®^ spectrophotometer, and a Qubit^®^ 2.0 Fluorometer. The qualified RNA samples were submitted to Sangon Biotech Co., Ltd. (Shanghai, China) for library construction and sequencing. Briefly, Illumina sequencing libraries were constructed from 1 μg of total RNA using the VAHTS™ mRNA-seq V2 Library Prep Kit following the manufacturer’s protocol, which involved mRNA enrichment, fragmentation, complementary DNA (cDNA) synthesis, end repair, adapter ligation, size selection, and PCR amplification. The final libraries were evaluated for quality using an Agilent 2100 Bioanalyzer and subsequently sequenced on an Illumina NovaSeq platform in paired-end mode. The quality of raw reads was initially assessed using FastQC. Subsequently, Trimmomatic was employed to filter the data by removing adapters, trimming low-quality bases (Q-score < 20), performing sliding window trimming (window size: 5 base pairs [bp]), and discarding reads shorter than 35 nucleotides (nt). High-quality clean reads were thus retained for downstream analysis. Clean reads were aligned to the *Gallus gallus* reference genome [28] using HISAT2. The alignment quality and distribution uniformity were evaluated using RSeQC and Qualimap, while gene coverage was calculated with BEDTools. Gene expression levels were quantified using StringTie and normalized as Transcripts Per Kilobase Million (TPM). Differentially expressed genes (DEGs) were identified using DESeq2 with screening criteria of a *q*-value ≤ 0.001 and a fold change ≥ 2. Subsequently, Gene Ontology (GO) functional enrichment analysis was performed on the identified DEGs. Visualizations, including scatter plots, heat maps, and volcano plots, were generated using the ggplot2 package.

### Quantitative real-time PCR (qRT-PCR)

The expression levels of cytokine genes in splenocytes were determined by relative qRT-PCR. Genomic DNA was removed from the total RNA, and 1 μg of RNA was reverse-transcribed into cDNA. The resulting cDNA was diluted tenfold and stored at −20 ℃. RT-qPCR assays were performed using the Universal SYBR qPCR Mix (EnzyArtisan, Shanghai, China) on a Bio-Rad CFX384 Touch Real-Time PCR Detection System, following the manufacturer’s instructions. The primer sequences used for RT-qPCR are listed in Table 1. Relative gene expression levels were calculated using the $2^{-\Delta \Delta {\mathrm{C}}_\mathrm{t}}$ method, with GAPDH serving as the internal control.

**Table 1:** **The sequences of primers used for this study**

Gene	GenBank ID	Primer sequence (5′–3′)
*IL6*	NM_204628.2	F: AACAACCTCAACCTGCCCAA
		R: AGGTCTGAAAGGCGAACAGG
*IL21*	NM_001024835.2	F: AGAAAACCCTGGGGATGGATG
		R: TCACAGTTTTGGCGAATGTAGC
*TBX21*	XM_015299480.4	F: AACAACGTCGGGCAGATGAT
		R: GAACTGTGTCTCGGGGAAGG
*IL8L1*	NM_205498	F: GGCTGGAGCAAAAGGTATGG
		R: TTCCACATTCTTGCAGTGAGG
*IL8L2*	NM_205018.2	F: TGCTCTGTCGCAAGGTAGGA
		R: CTTGGCGTCAGCTTCACATC
*IL17F*	XM_426223.8	F: TTGACATTCGCATTGGCAGC
		R: GGGTCCTCATCGAGCCTGTA
*TNFSF15*	NM_001024578.2	F: AAGAGCACACCTGACAGTGAA
		R: GGTATCACCAGTGCGTTGCT
*HDC*	NM_001293288.2	F: CTGGCTGGCTAAAATGCTGG
		R: CACTCACAGTGCTCTGCAATA
*CD88*	NM_001079739.2	F: CACTGCATCACTCTAGGCTGT
		R: TGTAACAACAAGGCAGGCCA
*GAPDH*	NM_204305.2	F: GGACCAGGTTGTCTCCTGTG
		R: TCCTTGGATGCCATGTGGAC

### Statistical analysis

Data are presented as mean ± standard error of the mean (SEM), and individual data points are plotted. Statistical analyses and figure generation were performed using GraphPad Prism 10 software. Comparisons among multiple groups were conducted using one-way analysis of variance (ANOVA) followed by Tukey’s post hoc test. Statistical significance was defined as follows: *P < 0.05, **P < 0.01, ***P < 0.001, and ****P < 0.0001; ns indicates no significant difference. Statistical annotations in some figures have been simplified for clarity.

## Results

### Construction and characterization of the (H5 + H7) bivalent HA mRNA–LNP vaccine

**Figure 1:**
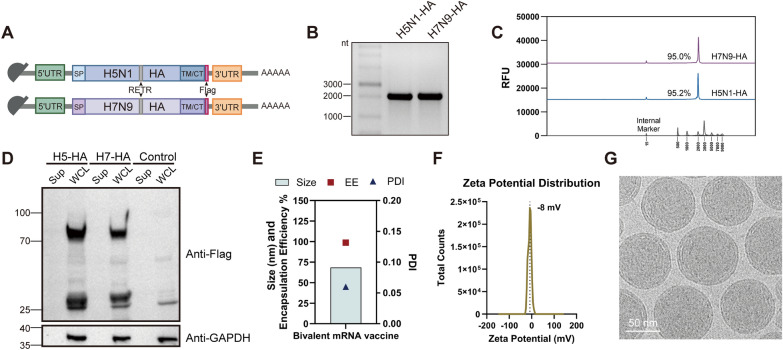
**Design, in vitro expression, and characterization of the bivalent mRNA–LNP vaccine**. **A** Schematic representation of the H5N1-HA and H7N9-HA mRNA constructs. The constructs comprise the 5′ UTR, a signal peptide (SP), the HA coding sequence, the transmembrane/cytoplasmic tail (TM/CT) fused with a C-terminal Flag tag, the 3′ UTR, and a poly(A) tail. The polybasic cleavage site within the HA gene was substituted with a “RETR” motif. Integrity analysis of the purified mRNAs was performed. The in vitro transcribed H5N1-HA and H7N9-HA mRNAs were analyzed by **B** agarose gel electrophoresis and **C** capillary electrophoresis. **D** Western blot analysis of the cell culture supernatant (Sup) and whole cell lysate (WCL) from BHK-21 cells transfected with H5-HA or H7-HA mRNAs. **E** Physicochemical characterization showing the mean particle diameter (size), encapsulation efficiency (EE%), and polydispersity index (PDI) of the bivalent mRNA–LNP. **F** Zeta potential distribution of the bivalent mRNA–LNP vaccine. **G** Cryo-EM image of the bivalent mRNA–LNP vaccine. Scale bar = 50 nm.

Two nucleoside-modified mRNA constructs encoding the HA proteins of HPAIV H5N1/B1417 (clade 2.3.4.4b) and H7N9/363–4 (group y.2.4) were generated. To enhance biosafety and antigen stability, the polybasic cleavage sites in both HA sequences were replaced with a monobasic cleavage site (RETR). The HA coding sequences were optimized on the basis of the Codon Adaptation Index (CAI) to improve expression efficiency. As depicted in Figure 1A, the mRNA constructs incorporate 5′ and 3′ UTRs, a Flag tag sequence, and a poly(A) tail. The integrity of the IVT mRNAs was assessed using 1% TBE agarose gel electrophoresis and capillary electrophoresis. As revealed by agarose gel electrophoresis, both mRNA constructs appeared as distinct, single bands, with no visible degradation smears or residual DNA template (Figure 1B). Consistently, capillary electrophoresis profiles exhibited clear and sharp peaks, corresponding to the expected size of approximately 2000 nucleotides (nt). Specifically, the integrity was determined to be 95.2% for H5-HA mRNA and 95.0% for H7-HA mRNA (Figure 1C). These results demonstrate the high purity and integrity of the synthesized mRNAs, qualifying them for downstream experiments. Subsequently, we evaluated the protein expression of the two mRNAs in BHK-21 and chicken DF-1 cells. Western blot analysis of BHK-21 and DF-1 cell lysates detected specific bands at approximately 70 kDa (Additional file 1A), corresponding to the expected size of the full-length HA-Flag fusion protein, confirming its expression in both transfected cell lines. The IFA yielded congruent results, revealing positive expression signals for both mRNAs in each cell type (Additional file 1B). Since we engineered the HA sequence to retain its membrane-anchoring domain, we further investigated its cellular localization. Western blot analysis comparing the cell culture supernatant (Sup) and whole cell lysate (WCL) revealed that the HA proteins were exclusively retained in the cell lysate and not secreted into the supernatant (Figure 1D). This membrane-anchored mode was visually and quantitatively corroborated by high-resolution confocal microscopy (Additional file 1C) and flow cytometry analysis (Additional file 1D), which demonstrated robust localization and surface display of both H5-HA and H7-HA on the cell membrane.

Subsequently, we developed a bivalent mRNA–LNP vaccine by co-encapsulating H5-HA and H7-HA mRNAs at a 1:1 mass ratio into LNPs. The physicochemical properties of the formulated bivalent vaccine were systematically evaluated. DLS analysis revealed a uniform particle size with an average diameter of approximately 68.4 nm and a PDI of 0.06 (Figure 1E), indicating a narrow size distribution and a stable formulation. Furthermore, the RiboGreen assay demonstrated an encapsulation efficiency exceeding 95% (Figure 1E). The zeta potential of the formulated LNP was approximately −8 mV, indicating a stable, slightly negative surface charge (Figure 1F). Furthermore, Cryo-EM imaging confirmed that the bivalent mRNA–LNPs possessed a uniform, spherical morphology with typical solid lipid core structures (Figure 1G). This high efficiency suggests that the vast majority of mRNA was successfully loaded into the LNPs, which is conducive to stable storage and efficient delivery.

### The bivalent mRNA vaccine induces potent humoral immunity in SPF chickens

**Figure 2:**
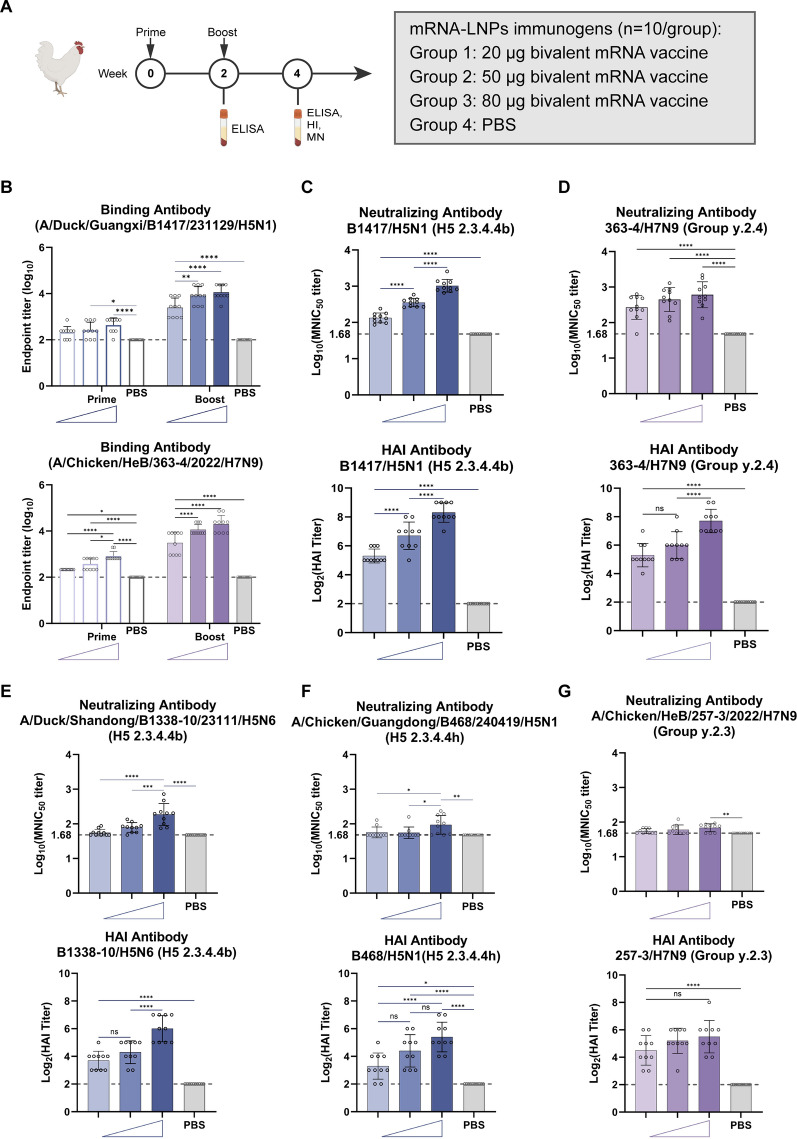
**Humoral immune responses induced by the bivalent mRNA vaccine in SPF chickens**. **A** Immunization and sampling schedule. Chickens were primed at week 0 and boosted at week 2. Serum samples were collected 2 weeks post-prime for ELISA assays, and 2 weeks post-boost, sera were collected for ELISA, HI, and MN assays. The study included four groups inoculated with varying doses of the bivalent vaccine (20, 50, or 80 μg) or PBS. Vaccine groups are indicated by triangles in the graphs below. **B** Specific IgY antibody titers against H5N1/B1417-rHA and H7N9/363–4-rHA in sera collected post-prime and post-boost, determined by ELISA. Neutralizing (upper panel) and HI (lower panel) antibody titers against the homologous B1417/H5N1 strain (**C**) and H7N9/363–4 strain (**D**) in post-boost sera. (**E**–**G**) Cross-neutralizing (upper panel) and HI (lower panel) antibody titers against heterologous H5N6/B1338-10 (**E**), H5N1/B468 (**F**), or H7N9/257–3 (**G**) strains in post-boost sera.

SPF chickens were immunized with the bivalent mRNA vaccine at three dosage levels (20, 50, and 80 μg) or a PBS placebo control, following a prime-boost regimen with a 2-week interval (as illustrated in Figure 2A). To evaluate the immunogenicity of the vaccine in SPF chickens, serum levels of antigen-specific IgY antibodies against homologous rHA antigens derived from H5N1/B1417 and H7N9/363–4 were measured by ELISA. Antigen-specific IgY responses were detectable in all vaccinated groups 2 weeks after primary immunization and exhibited a significant increase following booster vaccination. In addition, 2 weeks post-boost immunization, the endpoint titers of IgY antibodies in the three vaccine groups exhibited a dose-dependent increase and were significantly higher than those in the PBS control group, demonstrating the robust immunogenicity of the vaccine (Figure 2B). Subsequently, we further evaluated functional antibody responses in serum at 2 weeks post-boost using MN and HI assays. All vaccinated SPF chickens developed high titers of virus-neutralizing and HI antibodies against the vaccine-matched strains H5N1/B1417 and H7N9/363–4. Notably, these antibody responses exhibited a dose-dependent increase across the vaccine groups, with statistically significant differences observed in neutralizing antibody titers (Figure 2C and D). These results indicate that the bivalent mRNA vaccine elicits antibodies capable of effectively neutralizing homologous HPAIVs.

Importantly, we also evaluated the cross-reactive functional antibody responses against heterologous HPAIVs in chicken sera. When tested against the antigenically related H5N6/1338–10 virus belonging to the same clade 2.3.4.4b as the vaccine strain (Figure 2E), sera from chickens immunized with the 50-μg and 80-μg vaccine doses retained significant cross-neutralizing and HI activities. Similarly, neutralizing and HI antibodies against H5N1/B468, a heterologous virus belonging to clade 2.3.4.4 h (another prevalent clade in China), were also detectable in the high-dose vaccine groups (Figure 2F). Regarding the mismatched H7N9/257–3 strain (group y.2.3), neutralizing antibody titers were observed only in the 80-μg dosage group (Figure 2G). This suggests that the bivalent vaccine can induce effective immune responses against H7N9, although the neutralizing potency is attenuated likely due to significant antigenic divergence of the mismatched strains. Collectively, these data indicate that the immune response induced by the bivalent mRNA vaccine in SPF chickens demonstrates promising cross-protection potential against both clade-matched and mismatched heterologous H5 and H7 viruses.

### The bivalent mRNA vaccine elicits Th1-type cellular immunity

**Figure 3:**
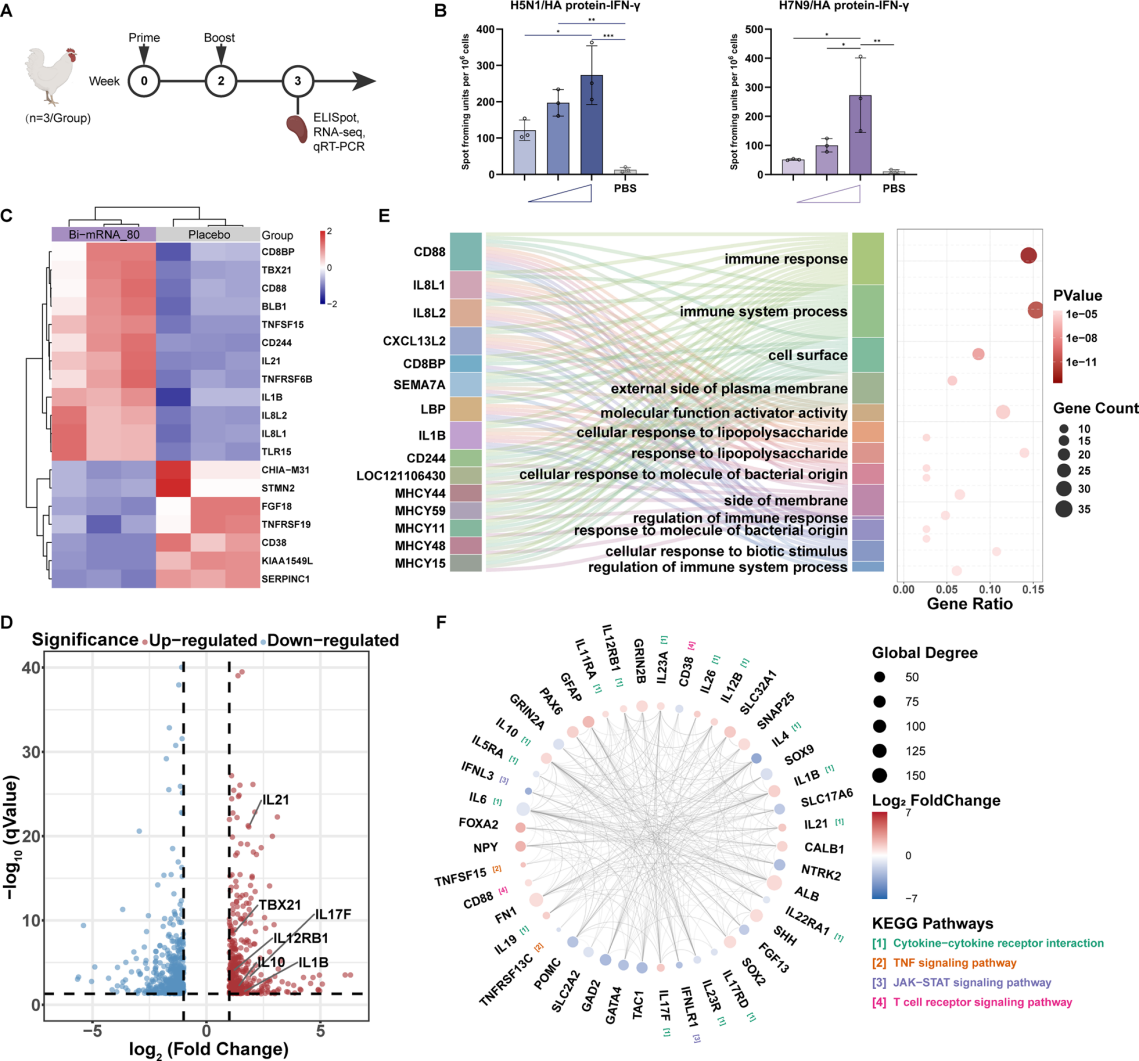
**Cellular immunity and transcriptomic profiling of the spleen in chickens following bivalent mRNA vaccination**. **A** Schematic representation of the immunization and sampling schedule. SPF chickens were primed at week 0 and boosted at week 2. Spleens were harvested at week 3 for ELISpot assays and RNA sequencing (RNA-seq). **B** Antigen-specific IFN-γ ELISpot responses against H5N1 and H7N9 rHA proteins. The bar graph displays the number of spot-forming units (SFU) per 10^5^ splenocytes in different dose groups compared with the PBS control. Data are presented as mean ± SEM. **C** Heat map analysis of DEGs in spleens of the vaccine group (Bi-mRNA_80) versus the PBS control (placebo) group. Red indicates upregulated gene expression, and blue indicates downregulated expression. (**D**) Volcano plot of DEGs. The *x*-axis represents (log_2_ fold change), and the *y*-axis represents −log_10_(*q*-value). Red and blue dots denote significantly upregulated and downregulated genes, respectively. **E** GO enrichment analysis. The visualization illustrates the association between genes and biological processes. (**F**) Chord diagram analysis showing the relationship between key DEGs and KEGG signaling pathways.

To assess cellular immunity, specifically the T cell response, which is pivotal for controlling avian influenza virus (AIV), we measured the secretion of the hallmark cytokine IFN-γ in chicken splenic lymphocytes via ELISpot upon stimulation with vaccine-matched rHA antigens. Compared with the PBS control group, splenocytes from the bivalent mRNA vaccine-immunized chickens exhibited a dose-dependent increase in IFN-γ spot-forming units upon stimulation with H5-rHA or H7-rHA proteins (Figure 3B). These results clearly demonstrate that vaccination successfully elicited specific Th1-type cellular immune responses against both H5 and H7 antigens.

### The bivalent mRNA–LNP vaccination triggers extensive splenic transcriptome reprogramming

To gain insights into the molecular mechanisms underlying vaccine-induced cellular immunity, we performed RNA sequencing (RNA-seq) on spleen samples collected from the 80-μg bivalent mRNA vaccine (Bi-mRNA_80) and PBS (placebo) groups 1 week post-boost. Comparative analysis of transcriptomic profiles revealed a substantial number of DEGs between the two groups. Notably, the vaccine group exhibited high interindividual consistency in gene expression patterns, as shown in Figure 3C. The volcano plot further illustrates the transcriptional changes (Figure 3D). Specifically, several key immune-regulatory genes, including *IL10*, *IL17F*, and *TBX21*, were significantly upregulated, suggesting robust immune cell recruitment and activation within the spleen. Gene Set Enrichment Analysis (GSEA) additionally corroborated the systemic activation of “immune system process” in the mRNA vaccine group (Additional file 2A). Consistently, functional enrichment analysis visualized via Sankey and bubble plots (Figure 3E) revealed that the upregulated genes in the Bi-mRNA_80 group were predominantly enriched in biological processes such as “immune response” and “immune system process” (Figure 3E, left). Key upregulated genes included *CD88*, *IL8L1*, *IL8L2*, *CXCL13L2*, *SEMA7A*, and multiple major histocompatibility complex (MHC)-related genes. These genes are primarily involved in cellular immune responses, cytokine activity, cell surface receptor signaling pathways, and antigen processing and presentation. Taken together, these results further underscore the synergistic activation of pathways pivotal for cellular immunity within the spleens of vaccinated SPF chickens.

To identify core regulators within this immune landscape, DEGs were mapped to the Search Tool for the Retrieval of Interacting Genes (STRING) interaction database [29]. Subsequently, a protein–protein interaction (PPI) network was constructed using the top 50 core genes, which were selected on the basis of their global degree of connectivity and immune relevance (Figure 3F). Topological analysis of the network revealed that these core genes formed highly interconnected clusters, suggesting that the vaccine triggers a tightly orchestrated immune response. Notably, the majority of these core immune nodes were significantly upregulated and exhibited high connectivity, indicating their pivotal role as hubs in the signal transduction network. Integration of KEGG pathway analysis revealed the activation of multiple key signaling pathways, including cytokine–cytokine receptor interaction, the TNF signaling pathway, and the T cell receptor signaling pathway (Figure 3F), which are crucial for orchestrating antiviral immunity. Furthermore, we selected representative genes for innate immune sensing (*IL1B*) and adaptive immunity (*IL21*, *TBX21*) as seed nodes (Additional file 2B–D). The PPI network analysis demonstrated that these core genes exhibited significant positive correlations with a vast number of downstream effectors involved in lymphocyte activation and inflammatory regulation. The *IL1B* network (Additional file 2B) revealed a specific gene cluster exhibiting significant positive correlations and synchronous upregulation (visualized as red nodes connected by solid lines). Notable genes in this cluster include *IL8L1*, *IL8L2*, and vascular cell adhesion molecule 1 (*VCAM1*), which play pivotal roles in neutrophil chemotaxis and vascular endothelial adhesion. These findings indicate that the bivalent mRNA–LNP vaccine formulation successfully induced a local inflammatory microenvironment, a necessary prerequisite for recruiting immune cells to the vaccination site. The *IL21*-centered network (Additional file 2C) unveiled its intimate connectivity with T cell co-stimulatory factors (e.g., *TNFSF15*) and molecules regulating cell adhesion and cytoskeletal dynamics (e.g., *ITGAD*, *TIAM1*, *MAP3K6*), indicating that during the induction of adaptive immunity, the vaccine actively facilitates T cell co-stimulatory signaling and enhances their tissue infiltration capacity. Notably, the *TBX21* network was not confined to canonical cytokine regulation but exhibited significant positive correlations with the complement chemotactic receptor *CD88* and the inflammation-regulating enzyme *HDC* (Additional file 2D). Furthermore, *TBX21* showed a positive correlation with the signal-transducing adaptor protein *STAP1*, while being negatively correlated with *MAPK10*. This suggests that the bivalent mRNA vaccine-induced *TBX21* may synergistically enhance the effector functions of the Th1 immune response by modulating the complement system interface and inflammatory mediators.

qRT-PCR validation was performed to ensure the reliability and reproducibility of the RNA-seq data. We selected ten representative DEGs known to be involved in immune regulation and inflammatory responses, including *IL21*, *TBX21*, *IL8L2*, *IL8L1*, *IL17F*, *TNFSF15*, *HDC*, *CD88*, and *IL6*. As shown in Additional file 2E, the expression patterns (log_2_ fold change) of these genes obtained from qRT-PCR were highly consistent with the RNA-seq results. Specifically, genes upregulated in the RNA-seq dataset also exhibited upregulation in the qRT-PCR analysis, while *IL6* showed downregulation in both methods. Furthermore, correlation analysis demonstrated a significant positive correlation between the two platforms (Pearson’s *r* = 0.8862, *P* < 0.01). These results confirm the accuracy of the RNA-seq quantification and support the validity of the bioinformatic analyses.

### The bivalent mRNA vaccine protects SPF chickens against homologous and heterologous H5/H7 virus challenges

Finally, to evaluate the protective efficacy of the bivalent mRNA vaccine in vivo, we challenged SPF chickens with either homologous or heterologous H5 or H7 virus strains. For the viral challenge, we selected the H5N1/B1417, H5N1/B468, and H7N9/257–3 strains, consistent with those used in the neutralization assays. The H7N9/363–4 strain was excluded because it failed to induce 100% mortality in the preliminary pathogenicity test. To assess the inhibition of early viral replication by the vaccine, we determined viral loads in lung tissues on 3 dpc. Following challenge with either homologous or heterologous H5N1 and H7N9 viruses, viral titers in the lungs of all three mRNA vaccine dosage groups were undetectable (below the limit of detection [LOD]), which was significantly lower than those observed in the PBS control group. These results demonstrate that the bivalent mRNA vaccine effectively restricts early viral replication in the lungs.

**Figure 4:**
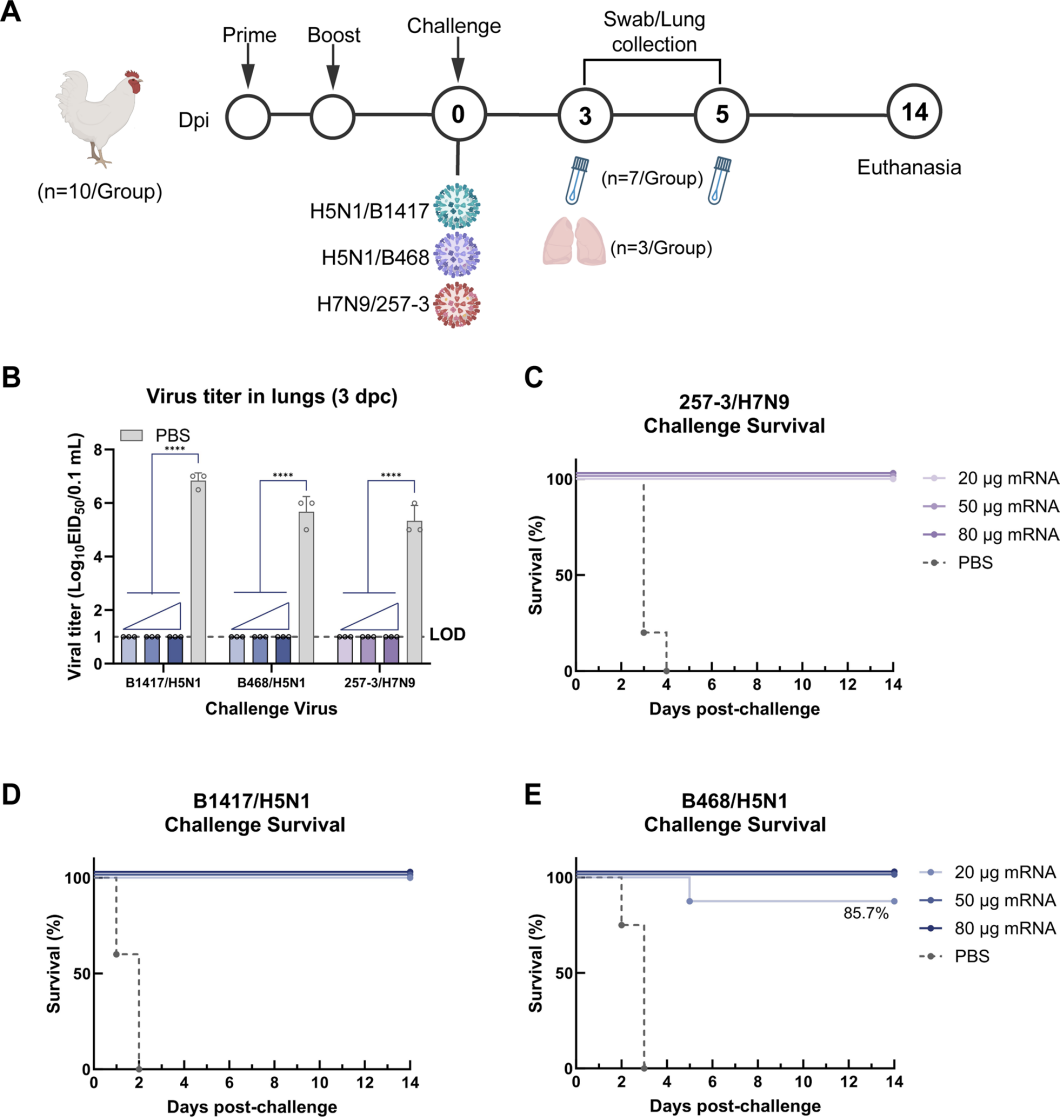
**Protective efficacy of the bivalent mRNA vaccine against HPAIV challenges in SPF chickens**. **A** Schematic of the experimental design. At 2 weeks post-boost, SPF chickens were challenged via the intranasal route with homologous H5N1/B1417, heterologous H5N1/B468, or H7N9/257–3 viruses. Oropharyngeal and cloacal swabs were collected at 3 and 5 dpc, and lung tissues were collected at 3 and 5 dpc. All surviving animals were euthanized at 14 dpc. **B** Viral titers in lung tissues at 3 dpc. Viral loads were titrated in embryonated chicken eggs and expressed as log_10_ EID_50_/0.1 mL. The dashed line indicates the limit of detection. Data are presented as mean ± standard deviation (SD). ****P < 0.0001 (compared with the PBS control group, determined by Tukey’s multiple comparison test). Survival curves of chickens following challenge with H7N9/257–3 (**C**), H5N1/B1417 (**D**), and H5N1/B468 (**E**).

In the homologous H5N1/B1417 challenge experiment, all SPF chickens in the PBS control group succumbed to infection within 2 dpc. In stark contrast, the immunized cohort exhibited a 100% survival rate (Figure 4D). Viral shedding was detected in oropharyngeal and cloacal swabs from the 20-µg and 50-µg vaccine groups at rates of 4/7 and 3/7 at 3 and 5 dpc, whereas it was undetectable in the 80-µg group. For the heterologous H5N1/B468 challenge, the PBS group experienced 100% mortality within 3 dpc. Although varying degrees of viral shedding were observed in the 20-µg, 50-µg, and 80-µg groups—with rates of 5/7, 3/7, and 1/7, respectively—the 50-µg and 80-µg dosages notably maintained a 100% survival rate (Figure 4E). Although one chicken in the 20-µg group died unexpectedly, no viable virus was recovered from its lung tissues. Upon challenge with the heterologous H7N9/257–3 strain, all control chickens died by 4 dpc. Conversely, vaccination conferred complete protection, resulting in 100% survival across all dosage groups (Figure 4C). However, viral shedding was still detected in the 20-µg, 50-µg, and 80-µg groups at frequencies of 4/7, 4/7, and 2/7, respectively.

In summary, the bivalent mRNA vaccine significantly reduced—or even completely abolished—viral shedding via the respiratory and digestive tracts in a dose-dependent manner. More importantly, the vaccine conferred complete protection against lethal homologous challenge while demonstrating robust cross-protection efficacy against heterologous strains (Table 2).

**Table 2:** **Virus shedding of chickens challenged with HPAIV**

Groups	H5N1/B1417		H5N1/B468		H7N9/257–3	
	3 dpc	5 dpc	3 dpc	5 dpc	3 dpc	5 dpc
20 μg bivalent mRNA	4/7	4/7	5/7	5/6^a^	4/7	4/7
50 μg bivalent mRNA	3/7	3/7	3/7	3/7	4/7	4/7
80 μg bivalent mRNA	0/7	0/7	1/7	1/7	2/7	2/7
PBS	–	–	–	–	1/1^b^	–

^a^One chicken died unexpectedly; ^b^only a single chicken survived; “–” not applicable due to the death of chickens.

## Discussion

In recent years, the global persistence and evolution of HPAI H5 and H7 subtypes continue to impose substantial losses on the poultry industry. Moreover, these viruses constitute a formidable public health threat, driven by frequent interspecies spillover events [10, 30]. The high mutation rate of the AIV genome results in antigenic drift, which frequently compromises the protective efficacy of existing inactivated vaccines due to antigenic mismatches [31]. Additionally, the protracted production cycle of traditional egg-based platforms hinders the ability to meet demands for rapid mass deployment during pandemics. Consequently, there is an urgent need to develop novel vaccine platforms capable of rapid adaptation to emerging variants and inducing broad-spectrum protection [32]. In this study, we evaluated the immunogenicity, protective efficacy, and underlying molecular immune mechanisms of a nucleoside-modified H5/H7 bivalent mRNA–LNP vaccine in SPF chickens, demonstrating its substantial potential as a next-generation vaccine against HPAIVs.

The HA glycoprotein, the primary surface antigen of influenza viruses, mediates host cell receptor binding and membrane fusion, thereby serving as a prime target for neutralizing antibodies [33]. However, ensuring its conformational stability is prerequisite for eliciting potent neutralizing responses. Previous studies have demonstrated that, compared with soluble forms, membrane-anchored HA more faithfully recapitulates the native viral surface structure, thereby exposing a greater abundance of conformation-dependent neutralizing epitopes [34, 35]. In this study, we employed a molecular design strategy by engineering the HA cleavage site into RETR motif to enhance protein stability. In vitro validation confirmed the robust expression of the mRNA-encoded HA protein on the cell membrane (Figure 1 and Additional file 1). This membrane-anchored mode of antigen presentation is crucial for efficient B cell receptor (BCR) recognition and activation, and likely accounts for the high titers of IgY, neutralizing, and HI antibodies induced by our bivalent vaccine in SPF chickens (Figure 2).

The immunogenicity of mRNA vaccines is intrinsically linked to mRNA modifications and the physicochemical properties of the delivery vehicle. Unmodified mRNA possesses virus-like characteristics and is prone to recognition by innate immune receptors (e.g., TLR7/8) and cytosolic sensors, thereby triggering excessive inflammatory responses, particularly activation of the type I interferon signaling pathway [36], while concurrently inducing eIF2α phosphorylation and inhibiting protein translation [37]. Accordingly, balanced innate immune activation is considered a key determinant of mRNA vaccine performance [38, 39]. In this study, m1Ψ modification was employed to reduce innate immune sensing and enhance translation efficiency [40], while lipid nanoparticles provided delivery and adjuvant functions [38, 41]. Consistent with this strategy, PPI network analysis revealed a tight co-expression cluster involving *IL8L1*, *IL8L2*, and *VCAM1* post-immunization (Additional file 2B). Specifically, the upregulation of *VCAM1* indicates vascular endothelial activation, which, in concert with the release of *IL8* family chemokines, orchestrates the targeted recruitment of heterophils and antigen-presenting cells (APCs). Meanwhile, the concomitant upregulation of the complement receptor (*CD88*) and lipopolysaccharide-binding protein (*LBP*) further enhances the efficiency of APCs in recognizing and internalizing the vaccine lipid particles. Notably, the synchronous upregulation of the anti-inflammatory cytokine IL-10, coupled with low expression levels of the acute-phase marker IL-6 (Figure 3F), suggests the activation of a robust negative feedback mechanism. This regulatory loop ensures that the inflammatory response remains transient and localized, thereby averting a systemic cytokine storm while still providing the essential danger signals required to prime adaptive immunity. This finely tuned innate immune activation, coupled with the expression of T helper cytokines such as IL-21, facilitates the formation of germinal centers (GCs) and the subsequent production of high-affinity antibodies [38, 42, 43].

Another distinct advantage of mRNA vaccines is the simultaneous induction of both humoral and cellular immunity, a feature that derives from their ability to mimic the intracellular antigen processing mechanisms of natural viral infections [44]. Corroborating this, our ELISpot results confirmed a significantly higher frequency of antigen-specific IFN-γ-secreting cells in the spleens of immunized chickens compared with the control group (Figure 3B), demonstrating that the vaccine successfully elicited a cellular immune response. Moreover, despite the relatively low titers of cross-neutralizing and HI antibodies against the heterologous strains H5N1/B468 and H7N9/257–3, the bivalent mRNA vaccine conferred complete protection to SPF chickens against a lethal challenge. Notably, viral loads in the lungs of the vaccinated group were undetectable during the early stage of infection (Figure 4). Collectively, these findings suggest that the broad-spectrum protection likely arises from the synergistic effect of multiple immune mechanisms. Polyclonal antibodies may target both variable regions of the HA head and conserved epitopes within the stem domain, while transcriptomic data indicate activation of Th1-type cellular responses and cytotoxic T lymphocytes (CTLs), which are likely to contribute to viral clearance when neutralizing antibody levels are suboptimal. *TBX21*, the master regulator governing Th1 cell differentiation and IFN-γ production, was significantly upregulated (Figure 3D). Its tight linkage with downstream interferon-stimulated genes (ISGs) and T cell activation markers (*CD244*, *CD38*) provides compelling molecular evidence that the vaccine not only activated T cells but also specifically orchestrated a Th1-type adaptive immune response [45]. Furthermore, the significant upregulation of genes such as *CD8BP* and *TNFSF15* is equally instrumental, as they are directly involved in potentiating the cytotoxic function of CD8^+^ T cells and fostering the formation of memory T cells [45, 46]. Such a robust Th1-type immune milieu is indispensable for combating influenza, as Th1-derived cytokines effectively facilitate the eradication of intracellular viral infections. However, it is important to note that despite the efficient viral suppression in the lungs, viral shedding was still detected in oropharyngeal and cloacal swabs. This observation reflects an inherent limitation of current intramuscular mRNA–LNP vaccines: While capable of inducing robust serum IgG and systemic cellular immunity, they often fail to elicit sufficient secretory IgA (sIgA) at the mucosal surfaces of the respiratory and digestive tracts [22, 47, 48]. The absence of a potent mucosal barrier allows for limited viral replication in the upper respiratory tract and subsequent shedding. Future strategies should explore mucosal administration routes (e.g., intranasal delivery) or optimize LNP vectors to enhance mucosal homing, with the ultimate goal of achieving “sterilizing immunity” to block viral transmission.

Despite the promising broad-spectrum protection demonstrated by our bivalent mRNA vaccine, this study has certain limitations. Primarily, vaccine efficacy was evaluated in SPF Babcock chickens, whereas genetic background, particularly variations in the MHC across diverse commercial broiler and layer breeds, may impact antigen presentation efficiency and the magnitude of adaptive immune responses. In addition, commercial poultry flocks frequently harbor maternally derived antibodies (MDAs) or face complex co-infection pressures [49]. Therefore, further validation of the vaccine’s field efficacy is required. Encouragingly, however, previous studies have established that mRNA vaccines can elicit potent antigen-specific antibodies in mice even in the presence of pre-existing immunity [22], suggesting the platform’s potential resilience in real-world applications. To augment immune durability, recent research demonstrates that optimizing mRNA secondary structures or UTRs can extend intracellular half-life and prolong protein expression [24, 40, 50], offering a promising avenue for refining our vaccine formulation. Furthermore, to address the suboptimal cross-neutralization against heterologous H7N9 strains, future antigen design strategies could prioritize the conserved HA stalk domain or construct multi-epitope tandem antigens [51]. These approaches are anticipated to expand the breadth of coverage against highly divergent viral variants. Additionally, while the current formulations were stored at −80 ℃ to preserve integrity for this proof-of-concept study, comprehensive thermal stability profiling and the development of thermostable formulations (e.g., via lyophilization) remain essential next steps to circumvent cold-chain dependency and facilitate practical deployment in the poultry industry.

In summary, we successfully engineered and validated a nucleoside-modified bivalent mRNA–LNP vaccine candidate against HPAI H5 and H7 subtypes. This vaccine not only elicited potent humoral immunity and Th1-type cellular responses in SPF chickens but, crucially, also conferred complete protection against lethal challenge with both homologous and heterologous HPAI strains, while effectively blocking viral shedding. Furthermore, this study is the first to elucidate the extensive immune reprogramming mechanisms induced by mRNA vaccines in the avian spleen at the transcriptomic level, providing novel insights into the mode of action of nucleic acid vaccines in non-model species. Capitalizing on the mRNA platform’s inherent advantages of design flexibility, rapid scalability, and multivalent adaptability, the bivalent vaccine developed herein represents a promising strategy for combating future antigenic drift and interspecies transmission, thereby strongly supporting global influenza control efforts under the “One Health” framework.

## Supplementary Information

**Additional file 1**. **Validation of cellular expression and membrane localization of the mRNA-encoded HA antigens.** (A–B) Validation of HA protein expression. BHK-21 and DF-1 cells were transfected with the purified mRNAs. Protein expression was detected by (A) Western blot analysis using an anti-Flag antibody and (B) IFA using the influenza HA stem-directed broadly neutralizing antibody, MEDI8852. (C–D) Membrane localization of the HA protein. The cellular localization of the protein in mRNA-transfected BHK-21 cells was assessed using high-resolution confocal microscopy (C) and flow cytometry (D).

**Additional file 2**. **Functional enrichment and validation of splenic transcriptome data from chickens immunized with bivalent mRNA vaccines.** (A) GSEA targeting the Immune system process. (B-D) Interaction and regulatory network analysis of core immune genes (*IL1B*, *IL21*, and *TBX21*). Node color intensity represents the log_2_ fold change value, and connecting lines indicate correlations between genes. (E) Validation of RNA-seq data by qRT-PCR. The left bar graph compares the log_2_ fold changes of the Bi-mRNA_80 group versus the Placebo group obtained from RNA-seq (pink) and qRT-PCR (blue). Data are shown as mean ± SEM. A Pearson correlation analysis between RNA-seq and qRT-PCR data is presented on the right panel.

## Data Availability

The datasets are not publicly available at this stage due to their large fi le sizes and because they are part of an ongoing project with further results being prepared for publication. However, they remain available from the corresponding author upon reasonable request.

## Availability of data and materials

The datasets generated and/or analyzed during the current study are not publicly available due to their large file sizes and because they relate to further ongoing research expansion, but are available from the corresponding author on reasonable request.

## References

[1] Avian Influenza - WOAH - World Organisation for Animal Health. https://www.woah.org/en/disease/avian-influenza/. Accessed 2025–12–30

[2] Pardo-Roa C, Nelson MI, Ariyama N, Aguayo C, Almonacid LI, Gonzalez-Reiche AS, Muñoz G, Ulloa M, Ávila C, Navarro C, Reyes R, Castillo-Torres PN, Mathieu C, Vergara R, González Á, González CG, Araya H, Castillo A, Torres JC, Covarrubias P, Bustos P, van Bakel H, Fernández J, Fasce RA, Johow M, Neira V, Medina RA (2025) Cross-species and mammal-to-mammal transmission of clade 2.3.4.4b highly pathogenic avian influenza A/H5N1 with PB2 adaptations. Nat Commun 16:2232 40044729 10.1038/s41467-025-57338-z PMC11882949

[3] Peacock TP, Moncla L, Dudas G, VanInsberghe D, Sukhova K, Lloyd-Smith JO, Worobey M, Lowen AC, Nelson MI (2025) The global H5N1 influenza panzootic in mammals. Nature 637:304–313 39317240 10.1038/s41586-024-08054-z

[4] Caserta LC, Frye EA, Butt SL, Laverack M, Nooruzzaman M, Covaleda LM, Thompson AC, Koscielny MP, Cronk B, Johnson A, Kleinhenz K, Edwards EE, Gomez G, Hitchener G, Martins M, Kapczynski DR, Suarez DL, Alexander Morris ER, Hensley T, Beeby JS, Lejeune M, Swinford AK, Elvinger F, Dimitrov KM, Diel DG (2024) Spillover of highly pathogenic avian influenza H5N1 virus to dairy cattle. Nature 634:669–676 39053575 10.1038/s41586-024-07849-4 PMC11485258

[5] Mostafa A, Naguib MM, Nogales A, Barre RS, Stewart JP, García-Sastre A, Martinez-Sobrido L (2024) Avian influenza A (H5N1) virus in dairy cattle: origin, evolution, and cross-species transmission. mBio 15:e0254224 39535188 10.1128/mbio.02542-24 PMC11633217

[6] Zhu S, Harriman K, Liu C, Kraushaar V, Hoover C, Shim K, Brummitt SI, Limas J, Garvey K, McNary J, Gao NJ, Ryder R, Stavig B, Schapiro J, Morales C, Wadford DA, Howard H, Heffelfinger J, Campagna R, Iniguez-Stevens E, Gharibi H, Lopez D, Esbenshade L, Ptomey P, Trivedi KK, Herrera JA, Locke J, Moss N, Rzucidlo P, Hernandez K (2025) Human cases of highly pathogenic avian influenza A(H5N1) - California, September-December 2024. MMWR Morb Mortal Wkly Rep 74:127–133 40080512 10.15585/mmwr.mm7408a1 PMC11952288

[7] Liu Y, Chen Y, Yang Z, Lin Y, Fu S, Chen J, Xu L, Liu T, Niu B, Huang Q, Liu H, Zheng C, Liao M, Jia W (2024) Evolution and antigenic differentiation of avian influenza A(H7N9) virus, China. Emerg Infect Dis 30:1218–1222 38640498 10.3201/eid3006.230530 PMC11138980

[8] Yang L, Zhu W, Li X, Chen M, Wu J, Yu P, Qi S, Huang Y, Shi W, Dong J, Zhao X, Huang W, Li Z, Zeng X, Bo H, Chen T, Chen W, Liu J, Zhang Y, Liang Z, Shi W, Shu Y, Wang D (2017) Genesis and spread of newly emerged highly pathogenic H7N9 avian viruses in mainland China. J Virol 91:e01277-17 10.1128/JVI.01277-17 PMC5686710 28956760

[9] Global Human Cases with Influenza A(H5N1), 1997–2025. https://www.cdc.gov/bird-flu/php/surveillance/chart-epi-curve-ah5n1.html. Accessed 2026–1–1

[10] Shi J, Zeng X, Cui P, Yan C, Chen H (2023) Alarming situation of emerging H5 and H7 avian influenza and effective control strategies. Emerg Microbes Infect 12:2155072 36458831 10.1080/22221751.2022.2155072 PMC9754034

[11] Wu J, Ke C, Lau EHY, Song Y, Cheng KL, Zou L, Kang M, Song T, Peiris M, Yen H (2019) Influenza H5/H7 virus vaccination in poultry and reduction of zoonotic infections, Guangdong Province, China, 2017–18. Emerg Infect Dis 25:116–118 30355435 10.3201/eid2501.181259 PMC6302570

[12] Zeng X, Tian G, Shi J, Deng G, Li C, Chen H (2018) Vaccination of poultry successfully eliminated human infection with H7N9 virus in China. Sci China Life Sci 61:1465–1473 30414008 10.1007/s11427-018-9420-1

[13] Cui P, Shi J, Wang C, Zhang Y, Xing X, Kong H, Yan C, Zeng X, Liu L, Tian G, Li C, Deng G, Chen H (2022) Global dissemination of H5N1 influenza viruses bearing the clade 2.3.4.4b HA gene and biologic analysis of the ones detected in China. Emerg Microbes Infect 11:1693–1704 35699072 10.1080/22221751.2022.2088407 PMC9246030

[14] Zhang X, Yang Y, Han X, Wei D, Niu B, Huang Q, Li Y, Yin H, Zhang X, Liao M, Jia W (2025) Unique phenomenon of H5 highly pathogenic avian influenza virus in China: co-circulation of clade 2.3.4.4b H5N1 and H5N6 results in diversity of H5 virus. Emerg Microbes Infect 14:2502005 40326336 10.1080/22221751.2025.2502005 PMC12077465

[15] Sun W, Choy KT, Cheng KM, Brackman CJ, Cheng SM, Sit TH, Tse AC, Sims LD, Gu H, Tang AW, Wong AN, Tsang AT, Koo JC, Luk LL, Yen H, Peris M, Poon LL (2025) Detection and characterisation of high pathogenicity avian influenza virus (H5N1/H5N8) clade 2.3.4.4b, Hong Kong SAR, China, 2021 to 2024. Euro Surveill 30:2400839 39790075 10.2807/1560-7917.ES.2025.30.1.2400839 PMC11719804

[16] Hou Y, Deng G, Cui P, Zeng X, Li B, Wang D, He X, Yan C, Zhang Y, Li J, Ma J, Li Y, Wang X, Tian G, Kong H, Tang L, Suzuki Y, Shi J, Chen H (2024) Evolution of H7N9 highly pathogenic avian influenza virus in the context of vaccination. Emerg Microbes Infect 13:2343912 38629574 10.1080/22221751.2024.2343912 PMC11060016

[17] Corbett KS, Edwards DK, Leist SR, Abiona OM, Boyoglu-Barnum S, Gillespie RA, Himansu S, Schäfer A, Ziwawo CT, DiPiazza AT, Dinnon KH, Elbashir SM, Shaw CA, Woods A, Fritch EJ, Martinez DR, Bock KW, Minai M, Nagata BM, Hutchinson GB, Wu K, Henry C, Bahl K, Garcia-Dominguez D, Ma L, Renzi I, Kong W, Schmidt SD, Wang L, Zhang Y (2020) SARS-CoV-2 mRNA vaccine design enabled by prototype pathogen preparedness. Nature 586:567–571 32756549 10.1038/s41586-020-2622-0 PMC7581537

[18] Teo SP (2022) Review of COVID-19 mRNA vaccines: BNT162b2 and mRNA-1273. J Pharm Pract 35:947–951 33840294 10.1177/08971900211009650

[19] Fang E, Liu X, Li M, Zhang Z, Song L, Zhu B, Wu X, Liu J, Zhao D, Li Y (2022) Advances in COVID-19 mRNA vaccine development. Signal Transduct Target Ther 7:94 35322018 10.1038/s41392-022-00950-y PMC8940982

[20] Lu J, Tan S, Gu H, Liu K, Huang W, Yu Z, Lu G, Wu Z, Gao X, Zhao J, Yao Z, Yi F, Yang Y, Wang H, Hu X, Lu M, Li W, Zhou H, Yu H, Shan C, Lin J (2024) Effectiveness of a broad-spectrum bivalent mRNA vaccine against SARS-CoV-2 variants in preclinical studies. Emerg Microbes Infect 13:2321994 38377136 10.1080/22221751.2024.2321994 PMC10906132

[21] Corbett KS, Flynn B, Foulds KE, Francica JR, Boyoglu-Barnum S, Werner AP, Flach B, O’Connell S, Bock KW, Minai M, Nagata BM, Andersen H, Martinez DR, Noe AT, Douek N, Donaldson MM, Nji NN, Alvarado GS, Edwards DK, Flebbe DR, Lamb E, Doria-Rose NA, Lin BC, Louder MK, O’Dell S, Schmidt SD, Phung E, Chang LA, Yap C, Todd JM (2020) Evaluation of the mRNA-1273 vaccine against SARS-CoV-2 in nonhuman primates. N Engl J Med 383:1544–1555 32722908 10.1056/NEJMoa2024671 PMC7449230

[22] Furey C, Scher G, Ye N, Kercher L, DeBeauchamp J, Crumpton JC, Jeevan T, Patton C, Franks J, Rubrum A, Alameh M, Fan SHY, Phan AT, Hunter CA, Webby RJ, Weissman D, Hensley SE (2024) Development of a nucleoside-modified mRNA vaccine against clade 2.3.4.4b H5 highly pathogenic avian influenza virus. Nat Commun 15:4350 38782954 10.1038/s41467-024-48555-z PMC11116520

[23] Arevalo CP, Bolton MJ, Le Sage V, Ye N, Furey C, Muramatsu H, Alameh M, Pardi N, Drapeau EM, Parkhouse K, Garretson T, Morris JS, Moncla LH, Tam YK, Fan SHY, Lakdawala SS, Weissman D, Hensley SE (2022) A multivalent nucleoside-modified mRNA vaccine against all known influenza virus subtypes. Science 378:899–904 36423275 10.1126/science.abm0271 PMC10790309

[24] Wang Z, Tian C, Zhu J, Wang S, Ao X, He Y, Chen H, Liao X, Kong D, Zhou Y, Tai W, Liao M, Fan H (2025) Avian influenza mRNA vaccine encoding hemagglutinin provides complete protection against divergent H5N1 viruses in specific-pathogen-free chickens. J Nanobiotechnology 23:55 39881325 10.1186/s12951-025-03156-w PMC11776166

[25] Xu S, Zhang B, Yao J, Ruan W (2023) A new H9 influenza virus mRNA vaccine elicits robust protective immunity against infection. Vaccine 41:2905–2913 37005103 10.1016/j.vaccine.2023.03.049

[26] Kallewaard NL, Corti D, Collins PJ, Neu U, McAuliffe JM, Benjamin E, Wachter-Rosati L, Palmer-Hill FJ, Yuan AQ, Walker PA, Vorlaender MK, Bianchi S, Guarino B, De Marco A, Vanzetta F, Agatic G, Foglierini M, Pinna D, Fernandez-Rodriguez B, Fruehwirth A, Silacci C, Ogrodowicz RW, Martin SR, Sallusto F, Suzich JA, Lanzavecchia A, Zhu Q, Gamblin SJ, Skehel JJ (2016) Structure and function analysis of an antibody recognizing all influenza A subtypes. Cell 166:596–608 27453466 10.1016/j.cell.2016.05.073 PMC4967455

[27] Feng S, Huang M, Quan Y, Sun L, Lin J, Zhang D, Wei X, Wang Z, Ma X, Wang S, Pan Q, Jia W, Wei D, Feng G, Xu M, Deng Z, Zhang Q (2025) The spike 486 site is a key immune evasion point and a determinant of the immunogenicity of the RBD-dimer mRNA vaccine against SARS-CoV-2 variants. Virology 610:110612 40561863 10.1016/j.virol.2025.110612

[28] Gallus_gallus - Ensembl genome browser 115. https://www.ensembl.org/Gallus_gallus/Info/Index. Accessed 2026–5–19

[29] Szklarczyk D, Kirsch R, Koutrouli M, Nastou K, Mehryary F, Hachilif R, Gable AL, Fang T, Doncheva NT, Pyysalo S, Bork P, Jensen LJ, von Mering C (2023) The STRING database in 2023: protein-protein association networks and functional enrichment analyses for any sequenced genome of interest. Nucleic Acids Res 51:D638–D646 36370105 10.1093/nar/gkac1000 PMC9825434

[30] Rather MA, Gul I, Aman M, Hassan A, Nazki S, Koul PA, Potdar V, Ganai NA, Khan AA, Chikan NA, Abdul-Careem MF, Shabir N (2025) Understanding the pandemic potential of the avian influenza virus- key insights into pathogenesis, transmission, and host immunity. Arch Microbiol 207:185 40549164 10.1007/s00203-025-04371-7

[31] Possas C, Marques ETA, Oliveira A, Schumacher S, Siqueira MM, McCauley J, Antunes A, Homma A (2025) Highly pathogenic avian influenza: pandemic preparedness for a scenario of high lethality with no vaccines. Front Public Health 13:1613869 40740369 10.3389/fpubh.2025.1613869 PMC12307385

[32] Huang P, Sun L, Li J, Wu Q, Rezaei N, Jiang S, Pan C (2023) Potential cross-species transmission of highly pathogenic avian influenza H5 subtype (HPAI H5) viruses to humans calls for the development of H5-specific and universal influenza vaccines. Cell Discov 9:58 37328456 10.1038/s41421-023-00571-x PMC10275984

[33] Wu NC, Wilson IA (2020) Influenza hemagglutinin structures and antibody recognition. Cold Spring Harb Perspect Med 10:a038778 31871236 10.1101/cshperspect.a038778 PMC7397844

[34] Cui X, Vervaeke P, Gao Y, Opsomer L, Sun Q, Snoeck J, Devriendt B, Zhong Z, Sanders NN (2024) Immunogenicity and biodistribution of lipid nanoparticle formulated self-amplifying mRNA vaccines against H5 avian influenza. NPJ Vaccines 9:138 39097672 10.1038/s41541-024-00932-x PMC11298010

[35] Gao D, Chen Y, Han D, Qi Q, Sun X, Zhang H, Feng H, Wang M (2017) Membrane-anchored stalk domain of influenza HA enhanced immune responses in mice. Microb Pathog 113:421–426 29174687 10.1016/j.micpath.2017.11.025

[36] De Beuckelaer A, Pollard C, Van Lint S, Roose K, Van Hoecke L, Naessens T, Udhayakumar VK, Smet M, Sanders N, Lienenklaus S, Saelens X, Weiss S, Vanham G, Grooten J, De Koker S (2016) Type I interferons interfere with the capacity of mRNA lipoplex vaccines to elicit cytolytic T cell responses. Mol Ther 24:2012–2020 27506450 10.1038/mt.2016.161 PMC5154477

[37] Bérouti M, Wagner M, Greulich W, Piseddu I, Gärtig J, Hansbauer L, Müller-Hermes C, Heiss M, Pichler A, Tölke AJ, Witte G, Hopfner K, Anz D, Sattler M, Carell T, Hornung V (2025) Pseudouridine RNA avoids immune detection through impaired endolysosomal processing and TLR engagement. Cell 188:4880–4895 40580950 10.1016/j.cell.2025.05.032

[38] Alameh M, Tombácz I, Bettini E, Lederer K, Sittplangkoon C, Wilmore JR, Gaudette BT, Soliman OY, Pine M, Hicks P, Manzoni TB, Knox JJ, Johnson JL, Laczkó D, Muramatsu H, Davis B, Meng W, Rosenfeld AM, Strohmeier S, Lin PJC, Mui BL, Tam YK, Karikó K, Jacquet A, Krammer F, Bates P, Cancro MP, Weissman D, Luning Prak ET, Allman D (2021) Lipid nanoparticles enhance the efficacy of mRNA and protein subunit vaccines by inducing robust T follicular helper cell and humoral responses. Immunity 54:2877–2892 34852217 10.1016/j.immuni.2021.11.001 PMC8566475

[39] Pardi N, Hogan MJ, Porter FW, Weissman D (2018) mRNA vaccines - A new era in vaccinology. Nat Rev Drug Discov 17:261–279 29326426 10.1038/nrd.2017.243 PMC5906799

[40] Leppek K, Byeon GW, Kladwang W, Wayment-Steele KH, Kerr CH, Xu AF, Kim DS, Topkar VV, Choe C, Rothschild D, Tiu GC, Wellington-Oguri R, Fujii K, Sharma E, Watkins AM, Nicol JJ, Romano J, Tunguz B, Diaz F, Cai H, Guo P, Wu J, Meng F, Shi S, Participants E, Dormitzer PR, Solórzano A, Barna M, Das R (2022) Combinatorial optimization of mRNA structure, stability, and translation for RNA-based therapeutics. Nat Commun 13:1536 35318324 10.1038/s41467-022-28776-w PMC8940940

[41] Castaño D, Bettini E, Kumar B, Chudnovskiy A, Siv A, Protti G, Nakadakari-Higa S, Ceglia S, De Luna N, Chiu JE, Lederer K, Li SH, Ibrahim H, Muramatsu H, Mdluli T, Abraham E, Sahingur SE, Maillard I, Tam YK, Shin S, Hensley SE, Miner JJ, Lipinszki Z, Reboldi A, Pardi N, Spreafico R, Victora GD, Locci M (2025) Distinct components of mRNA vaccines cooperate to instruct efficient germinal center responses. Cell 188:7461–7480 41406961 10.1016/j.cell.2025.11.023 PMC12878702

[42] Lederer K, Castaño D, Gómez Atria D, Oguin THR, Wang S, Manzoni TB, Muramatsu H, Hogan MJ, Amanat F, Cherubin P, Lundgreen KA, Tam YK, Fan SHY, Eisenlohr LC, Maillard I, Weissman D, Bates P, Krammer F, Sempowski GD, Pardi N, Locci M (2020) SARS-CoV-2 mRNA vaccines foster potent antigen-specific germinal center responses associated with neutralizing antibody generation. Immunity 53:1281–1295 33296685 10.1016/j.immuni.2020.11.009 PMC7680029

[43] Pardi N, Hogan MJ, Naradikian MS, Parkhouse K, Cain DW, Jones L, Moody MA, Verkerke HP, Myles A, Willis E, LaBranche CC, Montefiori DC, Lobby JL, Saunders KO, Liao H, Korber BT, Sutherland LL, Scearce RM, Hraber PT, Tombácz I, Muramatsu H, Ni H, Balikov DA, Li C, Mui BL, Tam YK, Krammer F, Karikó K, Polacino P, Eisenlohr LC (2018) Nucleoside-modified mRNA vaccines induce potent T follicular helper and germinal center B cell responses. J Exp Med 215:1571–1588 29739835 10.1084/jem.20171450 PMC5987916

[44] Verbeke R, Hogan MJ, Loré K, Pardi N (2022) Innate immune mechanisms of mRNA vaccines. Immunity 55:1993–2005 36351374 10.1016/j.immuni.2022.10.014 PMC9641982

[45] Szabo SJ, Kim ST, Costa GL, Zhang X, Fathman CG, Glimcher LH (2000) A novel transcription factor, T-bet, directs Th1 lineage commitment. Cell 100:655–669 10761931 10.1016/s0092-8674(00)80702-3

[46] Provine NM, Larocca RA, Aid M, Penaloza-MacMaster P, Badamchi-Zadeh A, Borducchi EN, Yates KB, Abbink P, Kirilova M, Ng’Ang’a D, Bramson J, Haining WN, Barouch DH (2016) Immediate dysfunction of vaccine-elicited CD8+ T cells primed in the absence of CD4+ T cells. J Immunol 197:1809–1822 27448585 10.4049/jimmunol.1600591 PMC4991249

[47] Leonard RA, Spurrier MA, Skavicus S, Luo Z, Heaton BE, Spreng RL, Hong J, Yuan F, Heaton NS (2025) Development of DNA and mRNA-LNP vaccines against an H5N1 clade 2.3.4.4b influenza virus. J Virol 99:e0079525 40667976 10.1128/jvi.00795-25 PMC12363208

[48] Ykema MR, Davis MA, Kasal DN, Jennewein MF, Lo E, Singh J, Beaver S, Cross N, Melief E, Reed S, Press C, Brandt DS, McClary WD, Mohamath R, Fusco P, Bakken J, Casper C, Hartwig AT, Gerhardt A, Bowen RA, Voigt EA (2026) Intranasal replicon vaccine establishes mucosal immunity and protects against H5N1 and H7N9 influenza. Nat Commun 17:434 41530119 10.1038/s41467-025-64829-6 PMC12800107

[49] Maas R, Rosema S, van Zoelen D, Venema S (2011) Maternal immunity against avian influenza H5N1 in chickens: limited protection and interference with vaccine efficacy. Avian Pathol 40:87–92 21331952 10.1080/03079457.2010.541226

[50] Chaudhary N, Weissman D, Whitehead KA (2021) mRNA vaccines for infectious diseases: principles, delivery and clinical translation. Nat Rev Drug Discov 20:817–838 34433919 10.1038/s41573-021-00283-5 PMC8386155

[51] Yassine HM, Boyington JC, McTamney PM, Wei C, Kanekiyo M, Kong W, Gallagher JR, Wang L, Zhang Y, Joyce MG, Lingwood D, Moin SM, Andersen H, Okuno Y, Rao SS, Harris AK, Kwong PD, Mascola JR, Nabel GJ, Graham BS (2015) Hemagglutinin-stem nanoparticles generate heterosubtypic influenza protection. Nat Med 21:1065–1070 26301691 10.1038/nm.3927

