# Supplementary figures and images for: A bivalent mRNA–LNP vaccine confers broad-spectrum protection against both homologous and heterologous H5/H7 highly pathogenic avian influenza viruses in SPF chickens
Source: Vet Res. 2026 Jun 18;57:111. doi: 10.1186/s13567-026-01790-2 (PMC13281637; doi:10.1186/s13567-026-01790-2)

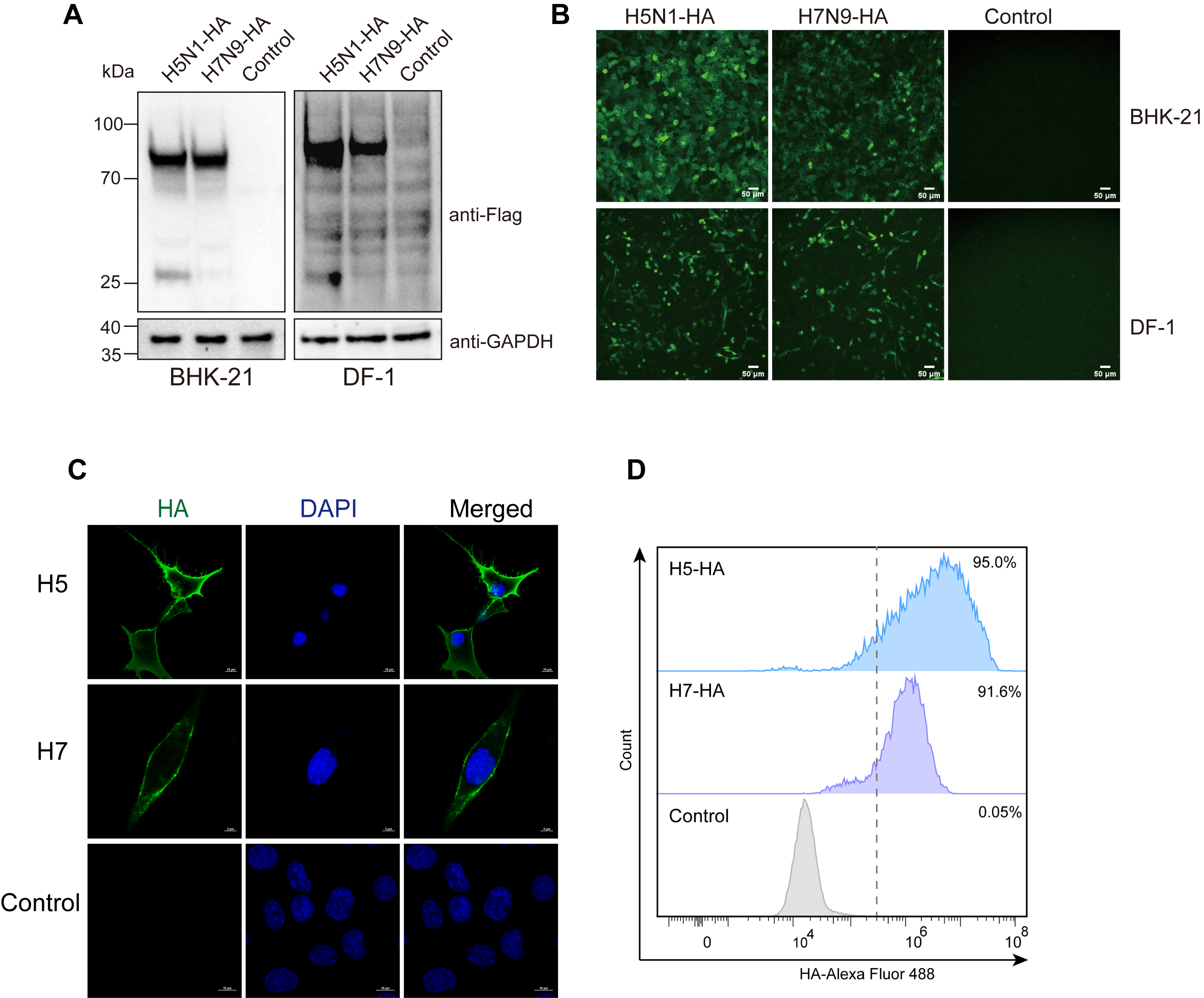

Supplement: Supplementary file 1 — Additional file 1. Validation of cellular expression and membrane localization of the mRNA-encoded HA antigens. (A–B) Validation of HA protein expression. BHK-21 and DF-1 cells were transfected with the purified mRNAs. Protein expression was detected by (A) Western blot analysis using an anti-Flag antibody and (B) IFA using the influenza HA stem-directed broadly neutralizing antibody, MEDI8852. (C–D) Membrane localization of the HA protein. The cellular localization of the protein in mRNA-transfected BHK-21 cells was assessed using high-resolution confocal microscopy (C) and flow cytometry (D). [file 13567_2026_1790_MOESM1_ESM.tif]

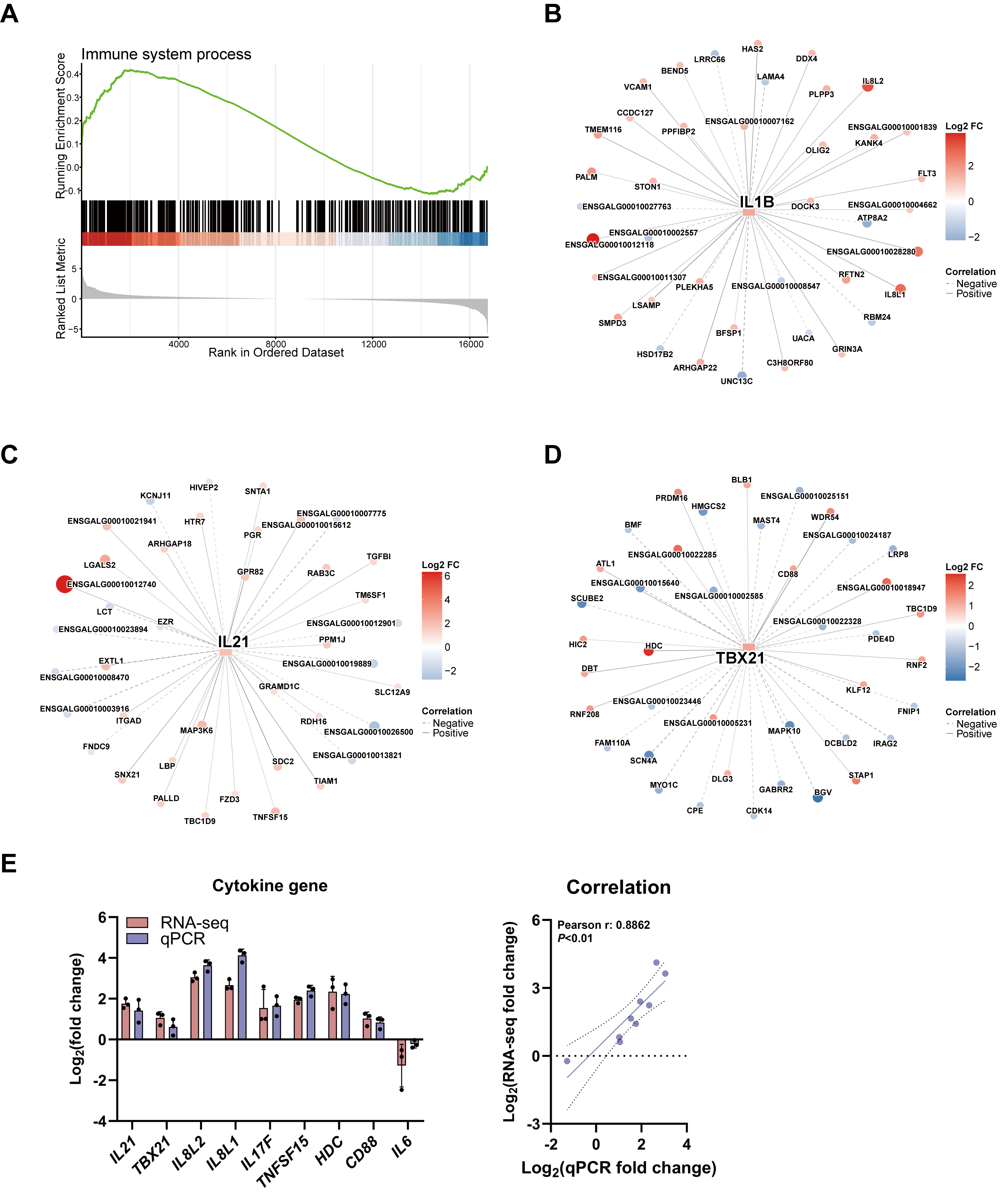

Supplement: Supplementary file 2 — Additional file 2. Functional enrichment and validation of splenic transcriptome data from chickens immunized with bivalent mRNA vaccines. (A) GSEA targeting the Immune system process. (B-D) Interaction and regulatory network analysis of core immune genes (IL1B, IL21, and TBX21). Node color intensity represents the log2 fold change value, and connecting lines indicate correlations between genes. (E) Validation of RNA-seq data by qRT-PCR. The left bar graph compares the log2 fold changes of the Bi-mRNA_80 group versus the Placebo group obtained from RNA-seq (pink) and qRT-PCR (blue). Data are shown as mean ± SEM. A Pearson correlation analysis between RNA-seq and qRT-PCR data is presented on the right panel. [file 13567_2026_1790_MOESM2_ESM.tif]
